# Tip-Enhanced Raman Imaging of Plasmon-Driven Coupling of 4-Nitrobenzenethiol on Au-Decorated Magnesium Nanostructures

**DOI:** 10.1021/acs.jpcc.3c01345

**Published:** 2023-04-12

**Authors:** Swati J. Patil, Vladimir Lomonosov, Emilie Ringe, Dmitry Kurouski

**Affiliations:** † Department of Biochemistry and Biophysics, Texas A&M University, College Station, Texas 77843, United States; ‡ Department of Materials Science and Metallurgy, University of Cambridge, 27 Charles Babbage Road, Cambridge CB3 0FS, United Kingdom; § Department of Earth Sciences, University of Cambridge, Downing Street, Cambridge CB2 3EQ, United Kingdom; ∥ The Institute for Quantum Science and Engineering, Texas A&M University, College Station, Texas 77843, United States

## Abstract

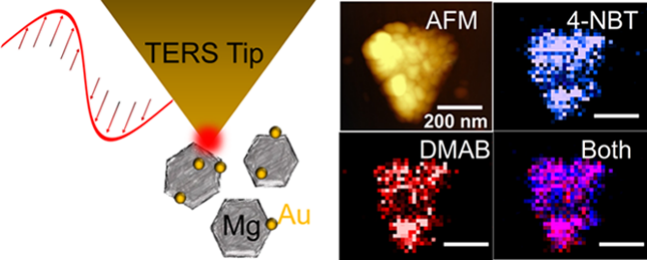

Magnesium nanoparticles (MgNPs) exhibit localized surface plasmon resonances across the ultraviolet, visible, and near-infrared parts of electromagnetic spectrum and are attracting increasing interest due to their sustainability and biocompatibility. In this study, we used tip-enhanced Raman spectroscopy (TERS) to examine the photocatalytic properties of MgNP protected by a thin native oxide layer and their Au-modified bimetallic analogs produced by partial galvanic replacement, Au-MgNPs. We found no reduction of 4-nitrobenzenethiol (4-NBT) to *p*,*p*′-dimercaptoazobisbenzene (DMAB) when a Au-coated tip was placed in contact with a self-assembled monolayer of 4-NBT molecules adsorbed on MgNPs alone. However, decorating Mg with Au made these bimetallic structures catalytically active. The DMAB signal signature of photocatalytic activity was more delocalized around AuNPs attached to Mg than around AuNPs on a Si substrate, indicating coupling between the Mg core and Au decorations. This report on photocatalytic activity of a bimetallic structure including plasmonic Mg paves the way for further catalyst architectures benefiting from Mg’s versatility and abundance.

## Introduction

Nanoparticles (NPs) of Au, Ag, and some other metals can sustain light-driven coherent oscillations of their conductive electrons called localized surface plasmon resonances (LSPRs).^1^ LSPRs enhance the local electric field and give rise to the electromagnetic enhancement in surface-enhanced Raman spectroscopy,^2^ a broadly used analytical technique that can detect surface-bound molecular analytes at the single-molecule level.^3,4^

Recently, the decay products of LSPRs have attracted much attention owing to their ability to turn light to nanoscopic local energy sources. Indeed, the rapid decay of the coherent oscillations generate a cascade of phenomena that includes energetic charge carriers (“hot carriers”, of which some are often called “hot electrons”) and heat.^5−7^ Hot carriers are short-lived highly energetic species that can populate unoccupied orbitals in molecules located on metal surfaces via indirect or direct charge transfer.^8,9^ Thus, hot carriers can contribute to and modify chemical reactions.^10^ Because of unequal rates of dissipation from metallic surfaces,^11,12^ the hot carriers with slower dissipation rates could create a steady-state charge on the metal surface that can alter rates and yields of plasmon-driven reactions.^13,14^ For instance, Yu and Jain reported that the yield of the plasmon-driven reduction of carbon dioxide on AuNPs could be altered by light intensity.^14^

The sharp Au-coated silicon tip of a tip-enhanced Raman spectroscopy (TERS) setup leads to a plasmonically enhanced local electric field^16−18^ that can be used not only for Raman scattering^19−21^ but also to catalyze surface chemical reactions.^10,22^ TERS can thus be used to both monitor and trigger chemical transformations on metallic surfaces.^11,15,23−25^ Two prominent reactions to probe plasmonic hot carrier effects are the reduction of 4-nitrobenzenethiol (4-NBT) to *p,p*′-dimercaptoazobisbenzene (DMAB)^22,26,27^ and the oxidation of 4-mercapto-phenyl-methanol (MPM) to 4-mercaptobenzoic acid (MBA).^15^ Li and Kurouski used them recently to measure the relationship between light intensity, rates, and yields of plasmon-driven reactions on mono- and bimetallic nanostructures, including Au@Pd microplates and nanoplates.^11,15,23^ For instance, the greater reaction rates on Au@Pd microplates than on Au microplates at the same light intensity have been attributed to the greater values of the rectified electric field on the surface of bimetallic nanostructures.^23^

Although AuNPs and some of their bimetallic analogs are good plasmonic materials, their broad utilization is limited by its relatively high cost and inability to sustain resonances in the ultraviolet (UV) and high-energy visible spectra.^28^ Cu, Al, and Mg have emerged as inexpensive alternatives, with the latter two allowing for UV LSPRs. CuNPs catalyzed the transformation of propylene to propylene oxide with improved selectivity compared to the thermocatalytic reaction.^29^ AlNPs decorated with catalytic metals have been shown to enhance the rate and selectivity of chemical transformations when illuminated with light.^30^ The plasmonic behavior of simple fabricated structures of Mg has been studied over the past 10 years,^31−33^ with more recent results on the resonances in sharp and faceted structures from colloidal syntheses.^34,35^ Asselin *et al.* also demonstrated that MgNPs can be partially replaced by another metal, *e.g.*, Au, Ag, or Pd,^36^ to form hybrid, decorated nanostructures that retain plasmonic properties. Lastly, we confirmed that the thin ∼10 nm oxide layer provides a barrier for further oxidation in air.^34,37^ MgNPs thus represent an exciting platform for light coupling, bimetallic nanostructure synthesis, and catalysis.

In this study, we used TERS to unravel the photocatalytic activity of both MgNPs and Au-decorated MgNPs (Au-MgNPs) in the plasmon-driven reduction of 4-NBT to DMAB. MgNPs were synthesized by a colloidal reduction of an organometallic Mg precursor, followed by partial galvanic replacement by HAuCl_4_, leading to heterogeneously decorated MgNPs as shown in Figure S1. Both types of NPs were first deposited on the Si substrate and then incubated in an ethanolic solution of 4-NBT to form a monolayer of 4-NBT on their surfaces. Next, we acquired TERS spectra from these nanostructures and performed TERS imaging, all with a 633 nm laser.

## Methods

### Chemicals

4-Nitrobenzenethiol (4-NBT), lithium pellets (99%), naphthalene (99%), 1.0 M di-*n*-butylmagnesium (MgBu_2_) in heptane, anhydrous tetrahydrofuran (THF), anhydrous isopropanol (IPA), polyvinylpyrrolidone (PVP) (average mol. weight 10,000), H_3_AuCl_4_ (99.99%), were purchased from Sigma-Aldrich. Anhydrous ethanol was purchased from Decon Labs. All chemicals were used as received without purification.

### Synthesis of MgNPs and Au-MgNPs

MgNPs were synthesized by the reduction of MgBu_2_ with lithium naphthalenide (LiNapht), as reported previously.^34^ Lithium (0.028 g), naphthalene (0.530 g), and PVP (0.020 g) were mixed with 10.75 mL of anhydrous THF in a 25 mL Schlenk flask under an Ar atmosphere and sonicated for 1 h, producing a dark green LiNapht solution. A total of 1.75 mL of MgBu2 in heptane was injected into lithium naphthalenide under vigorous stirring and left to react overnight at room temperature. The reaction mixture was quenched with anhydrous IPA. The solid product was recovered by centrifugation and then cleaned by repeated centrifugation and redispersion steps in anhydrous THF twice and anhydrous IPA twice, before redispersing in anhydrous IPA. The Mg content of the as-prepared Mg sample was determined by inductively coupled plasma mass spectrometry (ICP-MS). All glassware was washed with aqua regia (1:3 HNO_3_:HCl) and flame-dried under a vacuum before use.

Decoration of Mg NPs with Au was performed using an optimized protocol reported by Asselin et al.^36^ A total of 1 mL of the Mg NP suspension (0.50 mg/mL) was diluted with 2 mL of anhydrous IPA before the injection of 3 mL of H_3_AuCl_4_ solution (0.12 mg/mL) in anhydrous IPA. The mixture of Mg NPs with the Au precursor was left to react for 1 h in a sealed vial under stirring. The Au–Mg bimetallic nanostructures were recovered by centrifugation, and residual byproducts were removed by repeated (three times) centrifugation and redispersion in anhydrous IPA.

### Modification of the MgNPs and Au-MgNPs with 4-NBT

4-NBT was first dissolved in ethanol to reach the final concentration of 2 mM. A drop of MgNPs and Au-MgNPs from their stock solution was first deposited on Si wafers, which have been precleaned in piranha solution. Then, the Si wafer with MgNPs and Au-MgNPs was incubated in the 4-NBT solution for 2 h. The 4-NBT-modified MgNPs and Au-MgNPs on Si wafer were then rinsed thrice with ethanol to remove unbound 4-NBT molecules.

### TER Probe Fabrication

AFM tips were purchased from Appnano (Mountain View, CA). The tip parameters are force constant 2.7 N/m, resonance frequency 50–80 kHz, and amplitude 20 nm. The scanning rates for low- and high-magnification TERS images are 125 and 20 nm/s, respectively. For a metal deposition, AFM tips were placed in a thermal evaporator (MBrown, Stratham, NH). Metal deposition was conducted at ∼1 × 10^–6^ mbar by thermal evaporation of Au (Kurt J. Lesker, Efferson Hills, PA) at a 0.1 A/s rate to a final 70 nm Au thickness on the AFM tips. The temperature at the tip surface was ∼50 °C upon metal deposition.

### AFM-TER, SEM, HAADF-STEM, and STEM-EDS Imaging

Atomic force microscopy (AFM) and tip-enhanced Raman (TER) images were collected on an AIST-NT-HORIBA system equipped by a 633 nm continuous wavelength (CW) laser. Laser light was brought to the sample in a side-illumination geometry using a 100× Mitutoyo microscope objective. Scattered electromagnetic radiation was collected using the same objective and directed into a HORIBA iHR550 spectrograph equipped with a Synapse EM-CCD camera (HORIBA, Edison, NJ). SEM imaging of MgNPs sample drop-cast on Si wafers was performed on a Quanta-650F field emission gun scanning electron microscope (SEM), operated at 5 kV, and equipped with an Everhart–Thornley detector for secondary electron imaging. High-angle annular dark field scanning transmission electron microscopy (HAADF-STEM) and STEM-energy dispersive X-ray spectroscopy (STEM-EDS) images of Au-MgNPs drop cast on a Cu-supported lacey ultrathin carbon film were acquired at 200 kV on an FEI Osiris STEM equipped with a Bruker Super-X quad EDS detector.

## Results and Discussion

**Figure 1:**
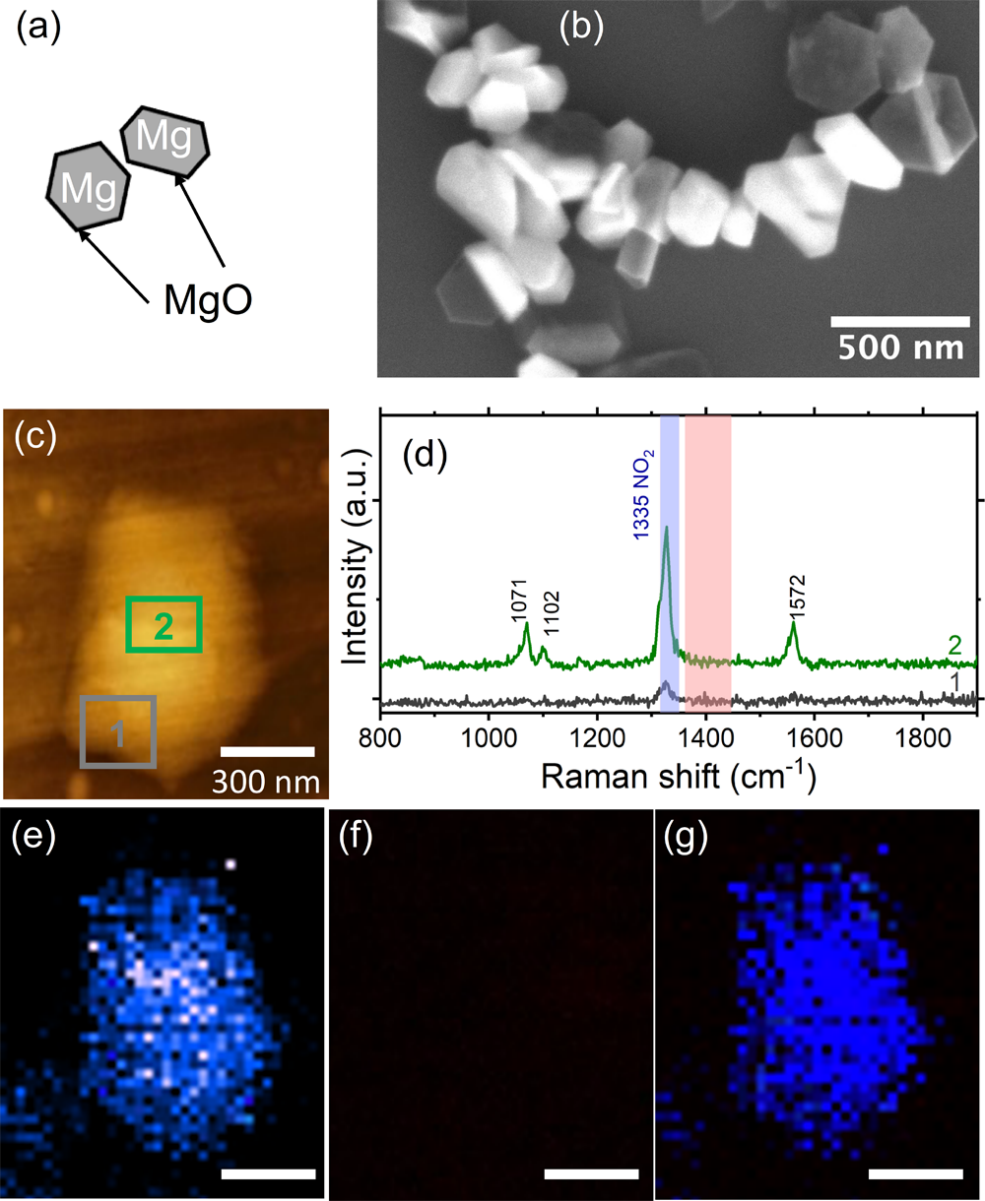
No reduction of 4-NBT to DMAB is observed over MgNPs. (a) Schematic of MgNPs covered by a thin oxide layer, (b) representative SEM image, (c) AFM image of a MgNP, (d) TER spectra from the two color-coded areas outlined in (c), (e) map of the 4-NBT band at 1335 cm^–1^ outlined in blue in (d), (f) map of the region around 1389 and 1432 cm^–1^, outlined in red in (d) showing no DMAB, and (g) overlay of the maps in (e) and (f). The scale bar for (c) applies to (e–g).

The TER spectrum of 4-NBT has three vibrational bands centered at 1073, 1335, and 1576 cm^–1^.^27^ The presence of these vibrational signatures of 4-NBT across MgNPs (Figure 1d and Figure S2) confirms its absorbance on the Mg surface. Surprisingly, TERS imaging did not reveal significant intensity variations between the center, corners, and edges of the studied MgNPs, unlike the field localization previously observed in metallic MgNPs from electron-energy loss spectroscopy^35,38^ and the position-dependent TERS enhancement of AuNPs, Cu nanowires, and Cu nanocubes.^39^ This is not ruling out Mg as a SERS/TERS material but rather is likely reflecting the lack of strong resonances at 633 nm in the large NPs observed as well as the potentially low or heterogeneous surface coverage of the analyte on the nonmetallic MgO surface. This low TERS enhancement was accompanied by the lack of detectable DMAB signal, which would, if present, display a doublet around 1390 and 1436 cm^–1^; no signal was detected even at higher light intensities of 30 to 1500 μW, at a wavelength of 633 nm. These results suggest that no reaction is occurring when 4-NBT is illuminated at 633 nm on bare MgNPs, possibly due to (i) the mismatch between the 633 nm excitation and the expected higher MgNP resonance wavelengths^38^ and (ii) the thin insulating oxide layer, characterized fully elsewhere,^34,35,38^ preventing any charge carrier from plasmon decay to reach the molecules. For the same latter reason, AlNPs alone have not been reported to be photocatalytically active; instead, AlNPs are decorated with a catalytically active metal and act as the plasmonic antenna in the antenna-reactor construct.^30^ Finally, although unlikely, suppressed plasmon-induced heating on MgNPs could be the cause of the suppressed reduction of 4-NBT to DMAB.^23^

**Figure 2:**
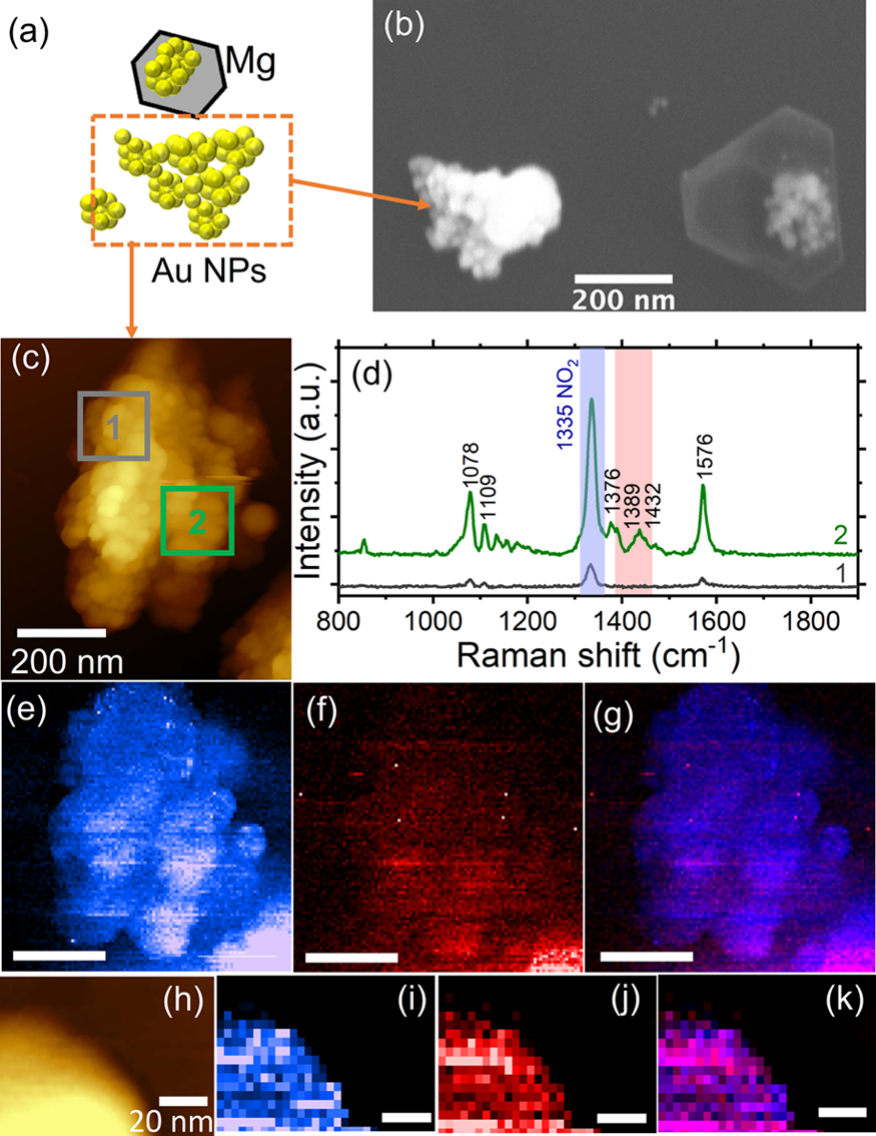
TERS measurements of monometallic Au nanostructures produced during partial galvanic replacement of MgNPs. Conversion of 4-NBT to DMAB is observed on AuNPs. (a) Schematic of AuNPs, (b) representative SEM image of AuNPs within the Au-decorated MgNPs, with the arrow pointing at typical Au aggregate containing no Mg, (c) AFM image of a AuNP aggregate, (d) TER spectra from the areas outlined and color-coded in (c), (e) map of the 4-NBT band at 1335 cm^–1^, (f) map of the 1389 and 1432 cm^–1^ region including bands from DMAB, and (g) overlay of the maps in (e) and (f). (h) AFM image of a different sample region also containing only AuNPs, (i) map of the 4-NBT band at 1335 cm^–1^, (j) map of the 1389 and 1432 cm^–1^ region including bands from DMAB, and (k) overlay of the maps in (i) and (j). The scale bar for (e–g) is 200 nm, and the scale bar for (h) applies to (i–k).

To enhance TERS signal and improve photocatalytic activity of Mg NPs, they were decorated with Au by partial galvanic replacement. However, in addition to bimetallic Au-decorated Mg NPs, galvanic replacement of Mg NPs was accompanied by formation of monometallic Au nanostructures (Figure 2a,b, Figure S1). Our results demonstrate that detached Au nanostructures enhance the TERS signals and demonstrate photocatalytic activity in 4-NBT reduction (Figure 2c–k). TER spectra and maps confirm previous results showing that plasmon-driven reduction of 4-NBT to DMAB occurs on Au surfaces.^23^ Furthermore, and importantly, here, significant TERS enhancement and DMAB signal were only observed on the AuNPs. A cliff-edge drop in both was obtained around the AuNPs (Figure 2e–g,i–k) and confirmed that our imaging setup had a spatial resolution sufficient to view the edges of the AuNPs.

**Figure 3:**
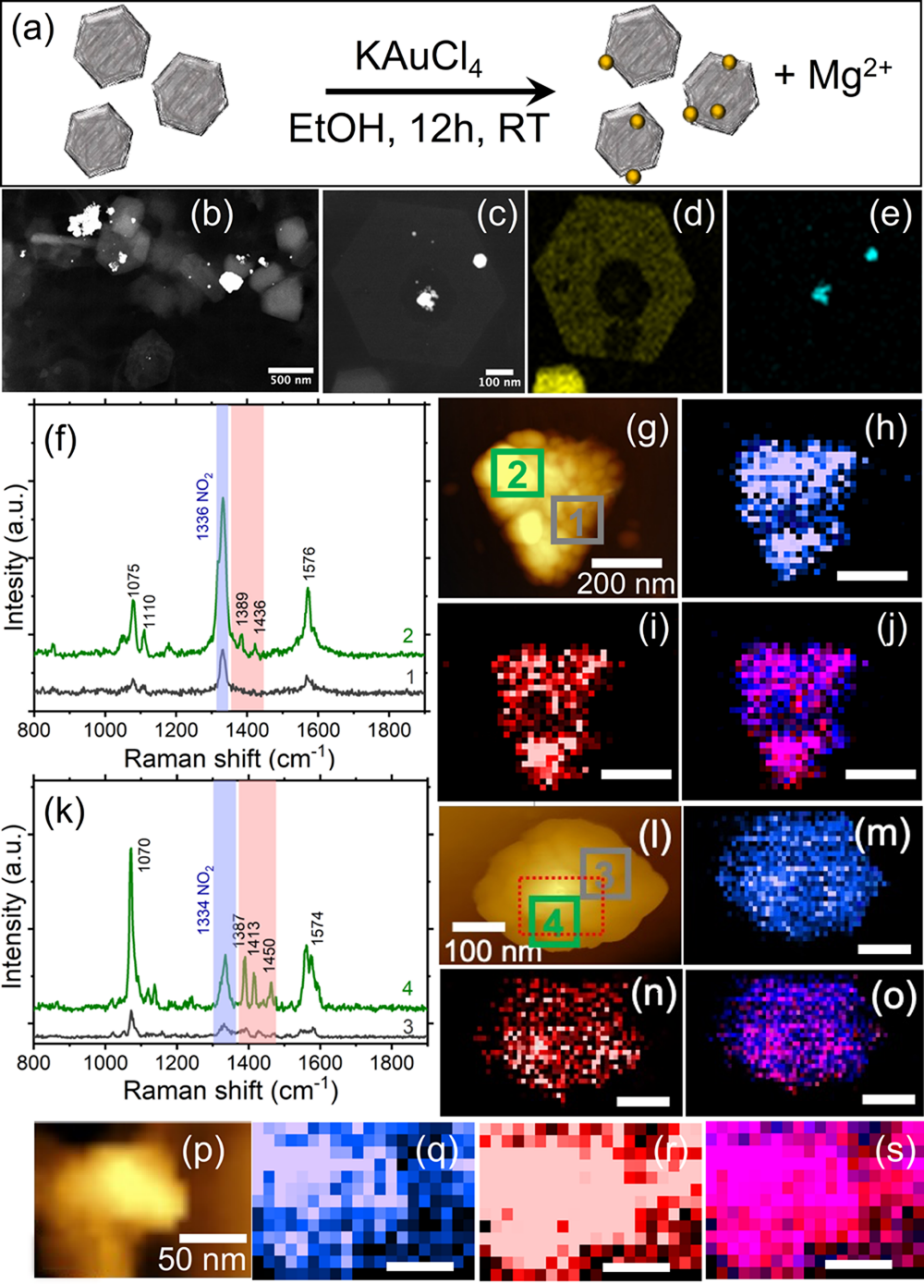
Conversion of 4-NBT to DMAB is observed across the entire structure for Au-decorated MgNPs. (a) Schematic of the partial galvanic replacement synthesis of Au-decorated MgNPs, (b, c) representative HAADF-STEM images of Au-decorated MgNPs (additional images in the SI), STEM-EDS maps of (d) Mg Kα and (e) Au M lines, (f) TER spectra from the areas outlined and color-coded (“1” and “2”) in the (g) AFM image of Au-decorated MgNPs, (h) map of the 4-NBT band at 1336 cm^–1^, (i) map of the 1389 and 1436 cm^–1^ region including bands from DMAB, and (j) overlay of the maps in (h) and (i) (scale bar = 200 nm). (k) TER spectrum of Au-decorated MgNPs at different areas outlined and color-coded (“1” and “2”) in the (l) AFM image of Au-decorated MgNPs, (m) map of the 4-NBT band at 1334 cm^–1^, (n) map of the 1387, 1413, and 1450 cm^–1^ regions including bands from DMAB, and (o) overlay of the maps in (m) and (n) (scale bar = 100 nm). (p) AFM image from the outlined region in (l), (q) map of the 4-NBT band at 1334 cm^–1^, (r) map of the 1387, 1413, and 1450 cm^–1^ regions including bands from DMAB, and (s) overlay of the maps in (q) and (r) (scale bar = 50 nm).

The formation of Au-decorated MgNPs by partial galvanic replacement was confirmed with high angle annular dark field scanning transmission electron microscopy (HAADF-STEM) and STEM-energy dispersive X-ray spectroscopy (STEM-EDS) (Figure 3b–e, Figure S1). Once decorated with Au, MgNPs (now Au-MgNPs) showed significant coupling with the excitation laser and photocatalytic reactivity. The TERS intensity of 4-NBT was much greater on Au-MgNPs than on MgNPs, attributable to an enhanced surface coverage and/or due to the increased electric field enhancement. The TERS enhancement of the 4-NBT signal was relatively uniform across the bimetallic structures, with slightly higher intensities found immediately atop AuNPs, hinting at Mg-Au plasmonic coupling leading to more delocalized enhancement. As expected from adding Au, DMAB was now produced on the Au-MgNPs. Excitingly, the Au islands did not behave as isolated NPs, and a coupling between the Mg and Au was observed (Figure 3, Figure S3). Specifically, we observed DMAB formation not only on the Au islands but also across the Mg/MgO surface around them, as shown in the DMAB TERS maps in Figure 3. This was unlikely caused by diffusion of DMAB, as sharp boundaries were observed on AuNPs alone (Figure 2) under similar imaging conditions, and the interactions of DMAB with MgO are expected to be similar to those on SiO_2_. The spread of the DMAB signal was also not produced by inadequate spatial resolution, as we established in Figure 2 that the TERS resolution was sufficient to see boundaries in small AuNPs. Therefore, we attribute the near-homogeneous DMAB production in Au-MgNPs to the plasmonic coupling of Mg and Au LSPRs that broaden spatially the enhanced electric fields and enable the reaction in a more delocalized manner around the AuNPs. It should be noted that different Mg@AuNPs exhibited unequal yield of DMAB on their surfaces (Figure 3,3k–of–j and ). The DMAB reaction yield is further evaluated with the ratio of reduction product to reactants of the TERS signal. The yield of DMAB on Au-MgNPs was 16.67%, whereas 20.94% of outlined locations on AuNPs observed the formation of DAMB (Figure S4). The yield of DMAB on AuNPs was found to be slightly higher than the yield of DMAB on Au-MgNPs.

## Conclusions

In conclusion, we used TERS to study the plasmon-mediated reduction of 4-NBT to DMAB. MgNPs did not produce a detectable level of DMAB, likely due to the low surface coverage of 4-NBT and the mismatch between the excitation and LSPR wavelengths. Single Au nanostructures demonstrated catalytic activity in 4-NBT reduction when illuminated with a 633 nm laser, as expected. When Mg and Au are brought together such that Au is present on the MgNPs, 4-NBT reduction was detected and the DMAB distribution was nearly homogeneous on the Mg surface, suggesting coupling between the two metals. This observation is a first step toward establishing Mg as a plasmonic material capable of taking part in plasmon-enhanced catalysis.

## Supporting Information Available

Additional HAADF STEM images, TER spectra, and maps on MgNPs and Au-MgNPs (PDF)
