# Supplementary material for: Tip-Enhanced Raman Imaging of Plasmon-Driven Coupling of 4-Nitrobenzenethiol on Au-Decorated Magnesium Nanostructures
Source: J Phys Chem C Nanomater Interfaces. 2023 Apr 12;127(16):7702–6. doi: 10.1021/acs.jpcc.3c01345 (PMC10359025; doi:10.1021/acs.jpcc.3c01345)
Supplement: Supplementary file 1 — jp3c01345_si_001.pdf [file jp3c01345_si_001.pdf]

Supporting Information for

Tip-Enhanced Raman Imaging of Plasmon-Driven Coupling of 4-Nitrobenzenethiol on Au-Decorated Magnesium Nanostructures

Swati J. Patil,[1] Vladimir Lomonosov,[2,3] Emilie Ringe[2,3*] and Dmitry Kurouski[1,4*]

1. Department of Biochemistry and Biophysics, Texas A&M University, College Station, Texas 77843, United States
2. Department of Materials Science and Metallurgy, University of Cambridge, 27 Charles Babbage Road, Cambridge, United Kingdom, CB3 0FS
3. Department of Earth Sciences, University of Cambridge, Downing Street, Cambridge, United Kingdom, CB2 3EQ
4. The Institute for Quantum Science and Engineering, Texas A&M University, College Station, Texas, 77843, United States

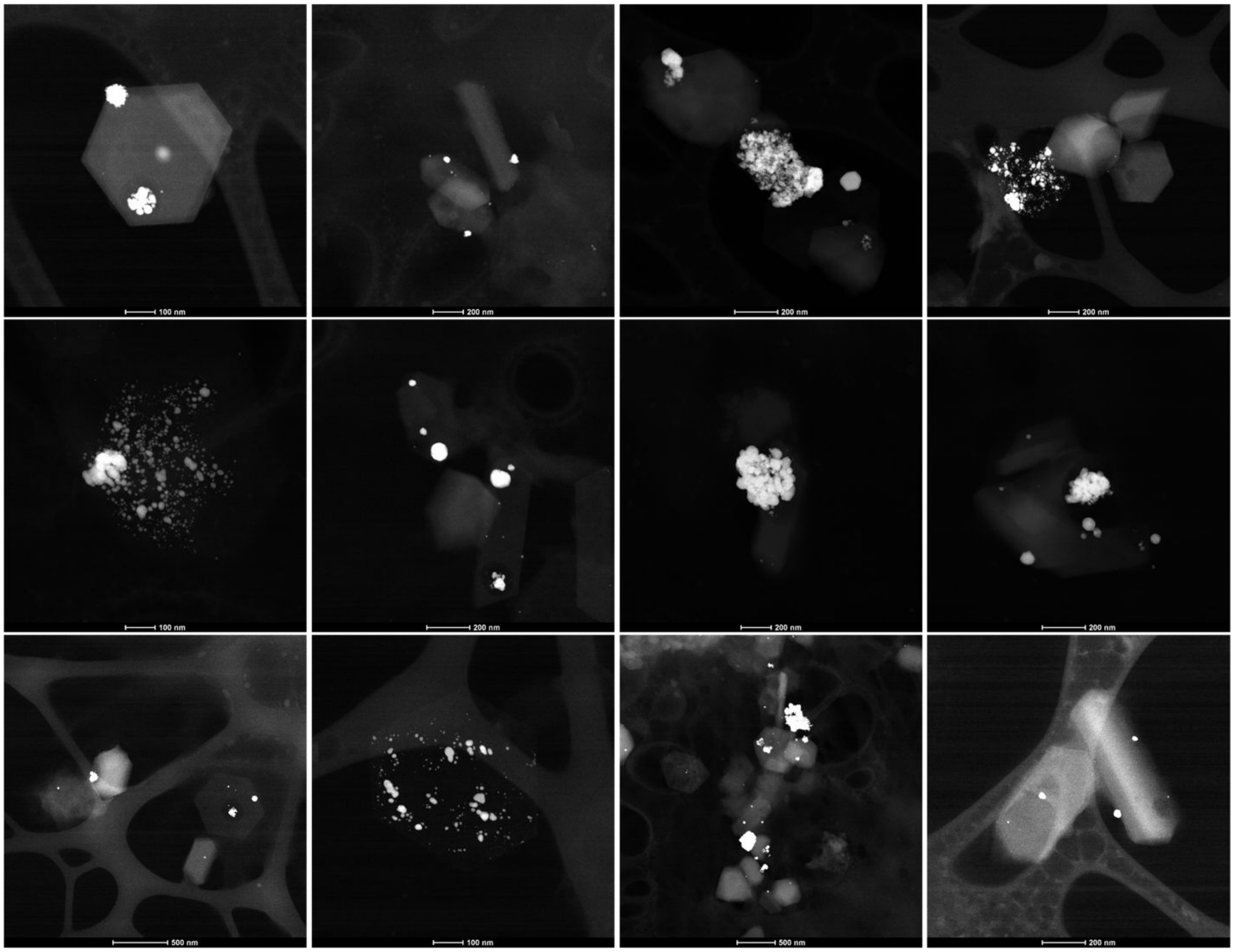
100 nm
200 nm
200 nm
200 nm
100 nm
200 nm
200 nm
200 nm
500 nm
100 nm
500 nm
200 nm

Figure S1. Additional HAADF-STEM images of Au-MgNPs showing the heterogeneity in decoration obtained by partial galvanic replacement.

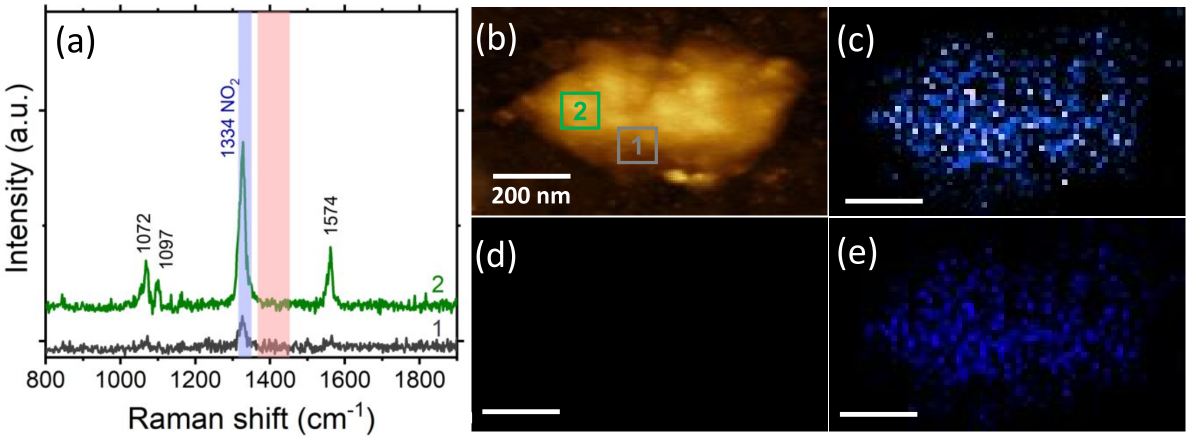

Figure S2. (a) Additional MgNPs TER spectra from the two color-coded areas outlined in (b), (c) map of the 4-NBT band at 1335 cm^{-1} outlined in blue in (a), (d) map of the region around 1389

and 1432 cm^{-1}, outlined in red in (a) showing no DMAB, and (e) overlay of the maps in (c) and (d). The scale bar in (b) applies to (b)-(e)

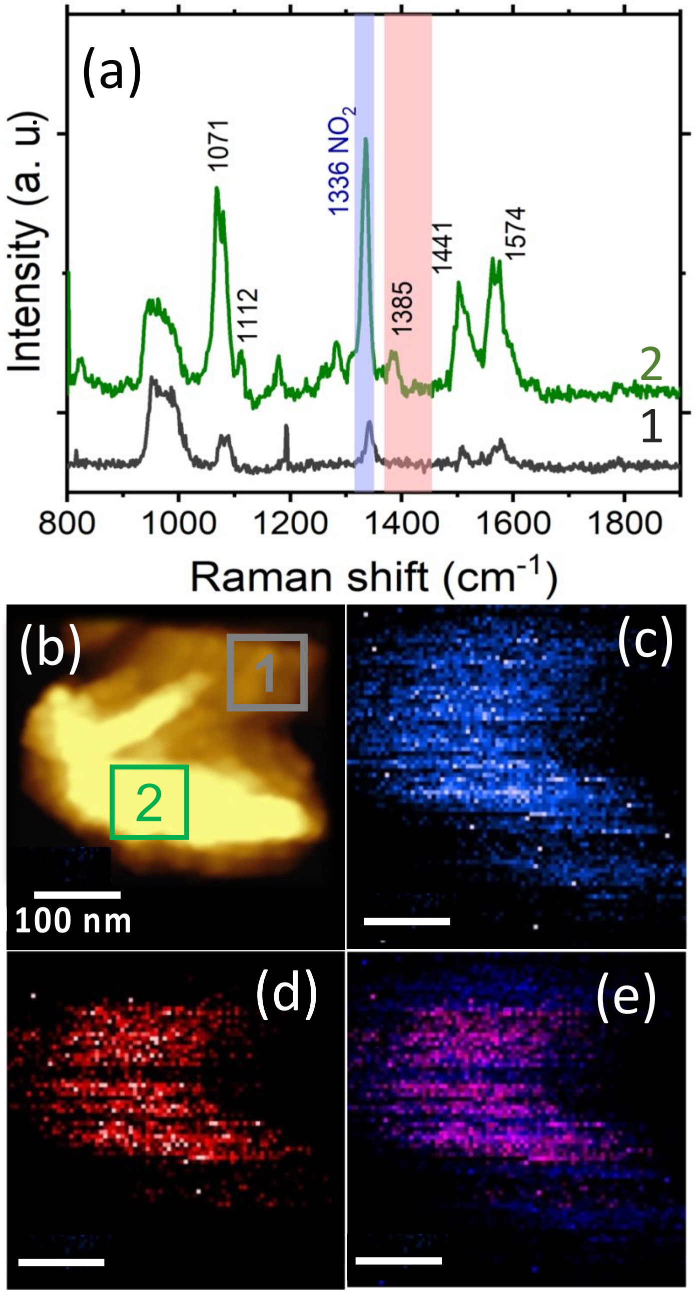

Figure S3. (a) Additional TER spectra from the areas outlined and color-coded in (b) the AFM image of an Au-decorated MgNPs, (c) map of the 4-NBT band at 1336 cm^{-1}, (d) map of the 1385, and 1441 cm^{-1} region including bands from DMAB, and (e) overlay of the maps in (c) and (d). The scale bar in (b) applies to (b)-(e).

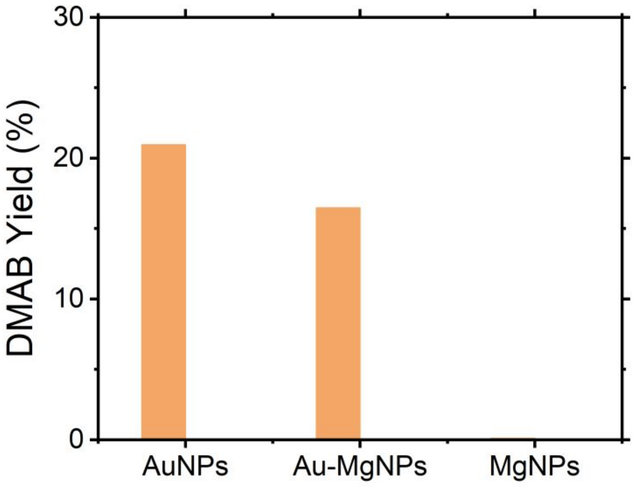

Figure S4. Histograms of the yield of DMAB on AuNPs, Au-MgNPs and MgNPs.
